# A Single-Institution Case Series of Outpatient Same-Day Mastectomy: Implementation of a Quality Improvement Project and Initiative for Enhanced Recovery After Surgery

**DOI:** 10.31486/toj.20.0040

**Published:** 2020

**Authors:** Mohamed-Aly Bakeer, Cameron Coker, Elisa Atamian, Daniel Yoo, Radbeh Torabi, Adam I. Riker

**Affiliations:** ^1^ Department of Surgery, Louisiana State University Health Sciences Center–New Orleans, New Orleans, LA; ^2^ Tulane University School of Medicine, New Orleans, LA; ^3^ Department of Surgery, Anne Arundel Medical Center, DeCesaris Cancer Institute, Annapolis, MD

**Keywords:** *Ambulatory surgical procedures*, *bupivacaine*, *mastectomy*, *nerve block*, *opioid-related disorders*, *pain management*

## Abstract

**Background:** National data demonstrate a trend toward outpatient same-day mastectomy. The possible drivers of this change include the costs related to hospital admission and effective management of postoperative pain. We retrospectively analyzed our single-institution experience with outpatient same-day mastectomy that incorporates a multimodal pain management regimen.

**Methods:** We retrospectively reviewed the medical records of patients who underwent same-day mastectomy at a single academic hospital. All patients received a multimodal, perioperative pain management regimen consisting of the intraoperative administration of 1,000 mg of intravenous (IV) acetaminophen and 30 mg of IV ketorolac, combined with the operating surgeon performing a 4- to 5-level, midaxillary, intercostal nerve block using liposomal bupivacaine. All patients were discharged with a prescription for acetaminophen with codeine, along with options for nonnarcotic alternatives as needed for pain.

**Results:** We reviewed the data on 72 patients who underwent mastectomies: 11 (15.3%) bilateral and 61 (84.7%) unilateral. The average age was 57 years, and average body mass index was 30 kg/m^2^. The average length of stay of 4 to 6 hours was a marked reduction compared to a 23-hour observational period or an inpatient hospital stay. The average follow-up was 20.1 weeks. Five patients presented to the emergency department (ED) within the 30-day postoperative period, with 2 patients (2.8%) requiring readmission to the hospital for non–pain-related issues. The other 3 patients (4.2%) were evaluated for specific pain-related issues but did not require admission and were discharged home from the ED.

**Conclusion:** Our data support outpatient same-day mastectomy incorporating a multimodal, perioperative pain management regimen as a safe and feasible treatment option. Potential additional benefits may include decreased oral opioid use and cost savings for the hospital.

## INTRODUCTION

During the 8-year time period between 2005 and 2013, the rate of unilateral and bilateral mastectomies increased by 2-fold and 5-fold, respectively.^1-2^ At the same time, mastectomy procedures began to shift to an outpatient setting. In 2003, approximately 22% of mastectomies across 17 states were performed in a hospital outpatient setting, and the number increased to approximately 42% by 2012.^1-2^ However, an outpatient procedure can include a 23-hour observation, an antiquated definition of an outpatient length of stay.

A wealth of data shows the safety of outpatient partial mastectomy (lumpectomy), with low reintervention rates, as well as reduced patient anxiety and depression compared to inpatient hospitalization for mastectomy.^3^ Susini et al also reported a high level of patient satisfaction and substantial financial savings for the hospital system with outpatient partial mastectomy.^3^ A 2009 systematic review by Marla and Stallard of 11 observational studies revealed a high rate of outpatient same-day surgeries for partial mastectomy, ranging from 86% to 100%, with 8 of the 11 studies examining readmission rates.^4^ In the 8 studies, the acute readmission rate was 0% for 6 studies, 7% for 1 study, and 8% for the remaining study.

Data from 2019 strongly suggest that such results can be applied to patients who are undergoing mastectomy, with 2 large studies definitively showing that same-day mastectomy is not only feasible but also offers significant advantages for patients in the home recovery setting.^5,6^ Patient advantages include improved physical and psychological recovery at home; increased independence, comfort, and control in the postoperative recovery period; and reduced risk of hospital-acquired infections. Outpatient same-day mastectomy can also have a significant positive impact on cost savings and optimization of hospital resources related to inpatient bed availability.

At our institution, outpatient same-day mastectomy incorporates a multimodal pain management regimen. Because of the public health awareness related to the misuse and potential abuse of prescription oral opioids, our unique pain management regimen was designed to provide the most effective and long-lasting perioperative and postoperative pain control possible. Given the high potential for opioid addiction and abuse in the postoperative setting, we wanted to minimize the amount and strength of prescribed oral opioids and limit the use of stronger oral opioids, such as oxycodone-acetaminophen (Percocet) and hydrocodone-acetaminophen (Norco).^7-13^

For this study, we wanted to determine if same-day mastectomy with the incorporation of a novel, multimodal pain management regimen is safe and feasible. If so, we planned to advance this approach throughout the health care system by developing an enhanced recovery after surgery (ERAS) clinical pathway and quality improvement (QI) project that could be applied across the United States in any breast surgery practice or hospital-based program.

## METHODS

We retrospectively reviewed the medical records of 72 consecutive patients undergoing mastectomy and the same perioperative pain management regimen. This study was formally approved by the Louisiana State University Health Sciences Center Institutional Review Board (IRB Protocol #10215). Patients received either unilateral (n=61) or bilateral (n=11) mastectomy performed by a single surgeon (A.I.R.) at a single institution between November 2015 and July 2017. Thirty patients had a unilateral mastectomy with a concomitant sentinel lymph node biopsy, 27 patients had a unilateral modified radical mastectomy only, and 4 patients had a unilateral modified radical mastectomy with a contralateral breast procedure (Table 1).

**Table 1.** Procedures Performed (n=72)

Procedure	n (%)
Total unilateral mastectomy	61 (84.7)
Unilateral simple mastectomy with a sentinel lymph node biopsy	30 (41.7)
Unilateral modified radical mastectomy only	27 (37.5)
Unilateral modified radical mastectomy with a contralateral breast procedure	4 (5.5)
Total bilateral mastectomy	11 (15.3)

All patients had a single, 15-French, fully fluted round drain placed along the inferior skin flap and extending into the axillary region for each breast for which a mastectomy was performed. For each case intraoperatively, the surgeon performed a multilevel, intercostal, midaxillary nerve block under direct visualization, using a total volume of 80 mL of liposomal bupivacaine (20 mL/bottle, diluted with 60 mL of normal saline). We used a 21-gauge needle or larger to avoid disrupting the liposomes, with the total volume of 80 mL aliquoted into four 20 mL syringes.

**Figure 1.**
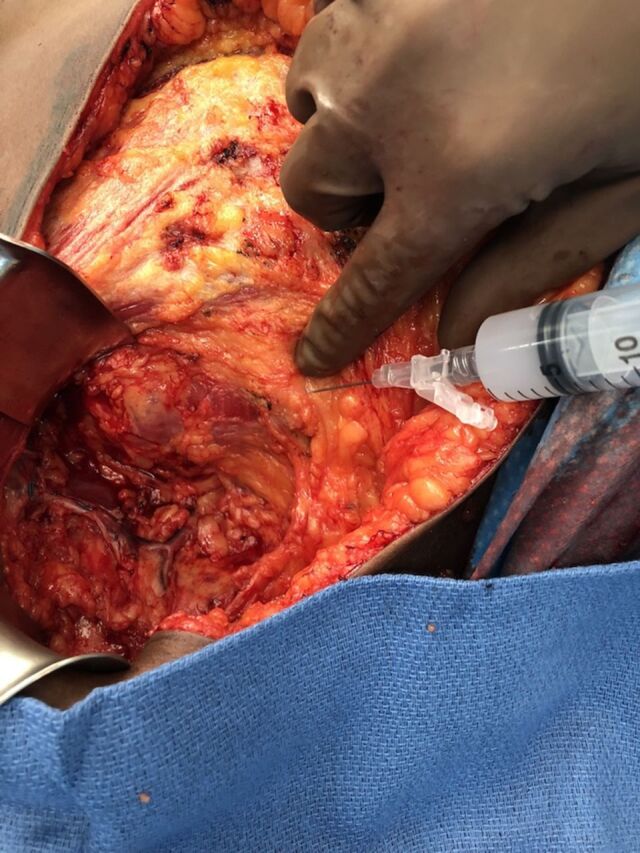
**For the intercostal block technique, the rib is palpated, the needle is directed toward the lower aspect of the rib, and the liposomal bupivacaine is directly injected into the neurovascular bundle just within the intercostal space. The needle cannot be advanced too deeply because of the potential for causing a pneumothorax. Approximately 5 mL of liposomal bupivacaine is injected at each rib level.**

Once the mastectomy and the axillary operation were completed, the wound was irrigated and hemostasis obtained. The single drain was then placed and sutured to the skin within the axilla for each breast for which a mastectomy was performed. The intercostal rib space within the midaxillary line and chest wall was identified and palpated. The needle was directed toward the inferior aspect of each rib, injecting approximately 5 mL of the liposomal bupivacaine into each rib neurovascular bundle. This process was repeated to the ribs above and below, so that a 4- to 5-level intercostal nerve block was completed (Figure 1). A remaining small amount of approximately 3 to 5 mL of the liposomal bupivacaine was infiltrated into the subcutaneous tissue around the drain site. For a bilateral mastectomy, the total volume was simply split in half, so that 40 mL total was used for each side.

**Figure 2.**
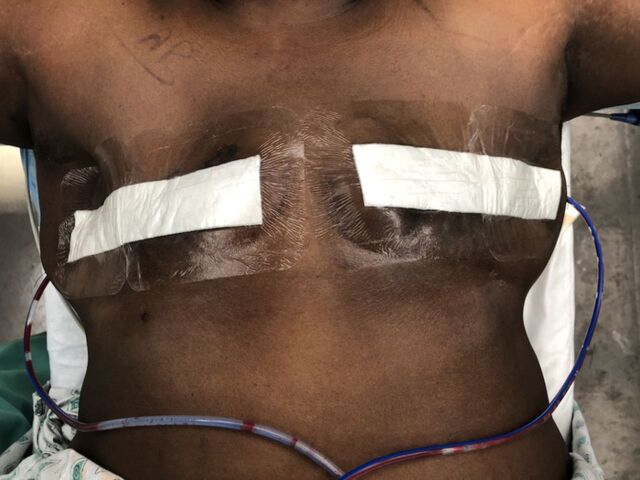
**Completed bilateral mastectomy incisions are covered with a small-width layer of a nonadherent dressing and an occlusive, water-impermeable transparent film dressing, with single, 15-French, fully fluted, round drains in place.**

As part of the multimodal pain regimen, we administered a single, intraoperative dose of intravenous (IV) acetaminophen 1,000 mg and the nonsteroidal anti-inflammatory drug (NSAID) ketorolac 30 mg. All patients received the same regimen of perioperative/postoperative pain management. All patients were allowed to shower the next day with the occlusive dressing in place. Our closure consists of completely absorbable sutures, with the third layer of closure comprised of liquid adhesive, such as a cyanoacrylate derivative. The mastectomy incision is covered with a small-width layer of a nonadherent dressing and an occlusive, water-resistant transparent film dressing (Figure 2).

Patients were discharged home from the postanesthesia care unit (PACU) the same day as their operation, usually within a 4- to 6-hour time period. All patients were given the option of either over-the-counter ibuprofen 600 mg, acetaminophen 1,000 mg, or a 6-day prescription for acetaminophen with codeine to be taken every 4 to 6 hours as needed for pain.

We examined patient demographics, type of mastectomy, tobacco use, and 30-day return to the emergency department (ED) and the reason for the visit.

## RESULTS

The average age of patients was 57 years, with an average body mass index of 30 kg/m^2^. The majority of patients were African American (66.7%) (Table 2).

**Table 2.** Patient Demographics and Characteristics (n=72)

Variable	Value
Mean age, years (range)	57 (31-73)
Mean body mass index, kg/m^2^ (range)	30 (18.9-45.5)
Ethnicity	
Caucasian/Hispanic/Latino	24 (33.3)
Unilateral mastectomy	21 (29.2)
Bilateral mastectomy	3 (4.2)
African American	48 (66.7)
Unilateral mastectomy	40 (55.6)
Bilateral mastectomy	8 (11.1)
Tobacco use	
Current tobacco user	17 (23.6)
Unilateral mastectomy	15 (20.8)
Bilateral mastectomy	2 (2.8)
Former tobacco user	14 (19.4)
Unilateral mastectomy	11 (15.3)
Bilateral mastectomy	3 (4.2)
Never smoker	38 (52.8)
Unilateral mastectomy	33 (45.8)
Bilateral mastectomy	5 (6.9)
Not documented	3 (4.2)

Note: Data are presented as n (%) unless otherwise noted.

The average follow-up for all patients was 20.1 weeks. Five patients (6.9%) presented to the ED within the 30-day postoperative period, and 2 (2.8%) required hospital readmission (Table 3). One patient was readmitted with symptoms consistent with a stroke, and the second patient developed a wound infection and abscess after 8 days. The other 3 patients (4.2%) were seen in the ED for pain-related issues, and all 3 were discharged home with a stronger prescription for oral opioids. All the other patients were seen in the clinic setting 10 to 14 days after the procedure for their first postoperative visit.

**Table 3.** Emergency Department (ED) Visits and Hospital Readmissions by Procedure Type (n=72)

Presentation	Procedure Type	n (%)
ED visit	Unilateral mastectomy	5 (6.9)
	Modified radical mastectomy	3 (4.2)
	Simple mastectomy	2 (2.8)
Hospital readmission	Unilateral mastectomy	2 (2.8)
	Modified radical mastectomy	2 (2.8)

## DISCUSSION

The overall number of patients undergoing mastectomy who are discharged home after an outpatient same-day procedure is increasing in the United States and Canada. This trend has helped to minimize inpatient hospital admission; maximize hospital bed utilization for sicker patients; decrease the overall total cost of care; and eliminate the potential for hospital-acquired infection, medication errors, and the potential for oversedating or overdosing with IV opioids.^4-6,14-24^ Goodman and Mendez were among the first to describe the consistent use of outpatient mastectomy, relying on a long-acting local anesthetic without epinephrine, specifically 0.5% bupivacaine hydrochloride, that was injected throughout the operative site into the surrounding soft tissues, as well as around the long thoracic, thoracodorsal, and intercostobrachial nerves.^25^ Other studies have reported high rates of patient satisfaction in conjunction with an organized and thorough patient education program focused on teaching patients how to manage and care for their drains at home.^14-25^

In 2019, Keehn et al reported that same-day mastectomy surgery using a perioperative care pathway and QI initiative was safe and well accepted in Alberta, Canada.^5^ The pathway was implemented in 13 hospitals across Alberta, with educational booklets, group classes, and other resources developed for providers, patients, and their families. After implementing this pathway, Keehn et al reported a substantial change in the percentage of patients undergoing same-day mastectomy, from 18/1,066 (1.7%) in 2001-2012 to 441/923 (47.8%) in 2018-2019.

Of note, the percentage of unplanned presentations of postoperative patients to the ED was high in the Keehn et al study, with 22% to 27% of patients presenting to the ED within 30 days of their operation during a 3-year time period. The unplanned hospital readmission rate was 3% to 6% during the same time period. However, neither metric was found to be statistically significant when compared to an overnight or longer hospital stay. Using same-day mastectomy as a QI initiative resulted in the release of an estimated 831 bed-days per year across the hospitals involved in the study. In voluntary patient reports of their experience with same-day mastectomy, approximately 90% of patients responded with “excellent or good” regarding the plans to go home, caring for themselves, drain care, incision care, and other support issues. Cost-estimated disparities were not evaluated in the study.^5^

In a similar fashion, Vuong et al initiated a pilot project across 21 medical centers in the Kaiser Permanente Northern California health care system to examine the feasibility and utility of same-day mastectomy, termed surgical home recovery (SHR).^6^ This large study included 84 surgeons performing 1,380 mastectomies, with 25% of patients undergoing bilateral mastectomy and 31% of patients having immediate implant-based reconstruction. Prior to implementing SHR, 23% of mastectomies were outpatient procedures, compared to 61% after the SHR implementation. This true change in clinical management was not associated with any significant differences in ED visits, reoperation, or hospital readmissions compared to an overnight hospital stay. Of note, the 5.2% rate of ED visits in the Vuong et al study was lower than the rate in the Keehn et al study and similar to our rate of 6.9%.

The Vuong et al SHR checklist provides a cursory overview of their pain management regimen, ranging from the availability of regional blocks (paravertebral, pectoralis), intraoperative administration of a lidocaine drip and IV acetaminophen, postoperative minimization of nausea with limited use of narcotics intraoperatively, and postoperative use of a scopolamine patch. Expectations were set with all patients preoperatively, with a focus on what to expect after surgery and on plans to minimize pain and discomfort, managed with nonopioid alternatives such as acetaminophen, NSAIDs, and local anesthetics such as arnica cream.^6^

Our single-institution experience with outpatient same-day mastectomy provides additional evidence and justification for implementing a QI project with an ERAS-driven protocol for same-day mastectomy. We believe that same-day mastectomy using a multimodal pain management regimen will result in a paradigm shift in our current care model for mastectomy patients across the United States. As mentioned previously, many of the outpatient mastectomy cases performed in prior years, and even today, are actually 23-hour hospital stays. Many patients stay beyond this time period, resulting in inpatient stays of 1 to 2 days in many cases. The studies by Keehn et al and Vuong et al strongly support a paradigm shift toward same-day mastectomy, ranging from 48% to 61%, which is most notable for the interval change toward a shift to same-day mastectomy with implementation of a QI protocol for pain management. Thus, a realistic endpoint for many organizations or surgeons may be to simply effect a positive net change in their practice patterns from a 23-hour observational stay toward same-day mastectomy. ED visits, postoperative complications, and readmission rates are all comparable to an inpatient hospital admission of at least 23 hours.^18^ The use of a long-acting bupivacaine infiltration has been shown to provide a marked opioid-sparing effect, especially within the first 48 hours following surgery.^26^

In terms of overall hospital costs, none of the studies cited in the discussion address potential cost savings of same-day mastectomy for the hospital or health care system. Early discharge after mastectomy has been described since the 1980s. Studies suggested the safety and economic advantage of discharge after 4 to 5 days as opposed to 9 to 10 days.^20-25^ Implicit in all of these studies, including ours, are the potential downstream cost savings derived from lower incidences of nosocomial infections, hospital overdosing with IV narcotics, wrongly administered medications, and the costs associated with having to manage these unexpected complications. Consideration should also be given to the cost differences associated with a same-day procedure and an average length of stay of approximately 4 to 6 hours vs a 23-hour observational period or longer.

Goodman and Mendez showed that an additional 2 to 3 days in the hospital added approximately $3,000 of cost for each patient.^25^ Another study found that the cost of an outpatient modified radical mastectomy was reduced by approximately 75% (approximately $5,000 per patient) when compared to the average 3-day inpatient stay.^21^ Little et al examined valuation and cost minimization with the use of liposomal bupivacaine in breast reconstructive surgeries and found a significant decrease in hospital length of stay, total costs, and rate of 30-day hospital readmission, suggesting a favorable economic profile.^27^ Little et al identified an estimated total cost savings of $11,510 per patient, with a direct cost savings of $6,399 per patient.

Some unique aspects of our single-institution experience with same-day mastectomy are worth noting. To the best of our knowledge, we are the first to report the incorporation of an intraoperative, surgeon-guided, multilevel rib/intercostal nerve block using liposomal bupivacaine as part of a perioperative multimodal pain management regimen. Liposomal bupivacaine appears to be the longest acting local anesthetic formulation available as of May 2020, with a range of duration of approximately 2 to 4 days based on our anecdotal experience with this study. The other aspects of the multimodal pain management regimen also appear to be unique, with no identified studies or citations of such a combination in the current (as of May 2020) literature.

This pain management regimen, combined with properly setting expectations and providing patient and family education, has translated into a safe, feasible, and highly successful approach to same-day mastectomy. In terms of postoperative oral opioid use, we have found that patients no longer need to be sent home with anything stronger than a few days’ prescription for acetaminophen with codeine. When given the option, many patients preferred no oral narcotics whatsoever, instead electing to take nonopioid alternatives, such as acetaminophen, ibuprofen, or another over-the-counter NSAID. However, we did not formally assess which patients chose which option in our retrospective review and cannot draw any firm conclusions as to the effectiveness of this pain management regimen.

We recognize some notable weaknesses of our study. We were unable to gather information related to patient-reported outcomes, such as overall patient satisfaction, postoperative pain levels each day, and comfort level with managing the drain at home. This information was not in the medical record in most instances; either it was not recorded or not discussed during the follow-up visits. Because our study was a retrospective review of prospectively followed patients, we did not provide patients with postoperative questionnaires addressing levels of pain or overall satisfaction. Therefore, we cannot draw conclusions as to the effectiveness of pain control or patient satisfaction beyond a patient's verbal anecdotal experience. Finally, we were unable to obtain accurate financial data related to cost savings associated with same-day mastectomy.

A few subtle but very important points are instrumental to the overall success of implementing a same-day mastectomy ERAS protocol. Once mastectomy is agreed upon as the recommended surgical approach, we discuss, in detail, all aspects of the patient's surgery with both the patient and family, focusing on what to expect after the operation is completed. We discuss the details of the multilevel intercostal nerve block, as well as the reasoning for why an overnight stay is not required with this approach. We further discuss and review the expected postoperative recovery experience, with the goal of a 4- to 6-hour total recovery before going home. The type of oral pain medication that the patient will be discharged home with is discussed, with nonopioid alternatives offered according to patient preference. We discuss the multiple advantages of going home the same day of surgery: decreased exposure to potential sources of hospital-based infections, not having to eat the hospital food, not being woken up in the middle of the night for vital signs and drain care, and sleeping in their own beds in the comfort of their homes. In addition, we discuss drain care management and range-of-motion exercises with every patient. An important part of this protocol is education of the patient and family members on how to properly manage the drain in the home setting.

The breast program nurse navigators at our center are instrumental with helping to educate patients and family members at several possible touch points throughout their experience. A patient may see a breast program nurse navigator prior to the scheduled operation, which is the preferred method in most cases. Sometimes, the patient will be seen again in the PACU and just prior to discharge to ask any last questions or review details about drain care. Additionally, our occupational and physical therapists see each patient and provide a booklet of range-of-motion exercises to perform daily at home.

## CONCLUSION

Our single-institution experience with outpatient same-day mastectomy, highlighting the use of a novel multimodal perioperative pain management regimen, shows that same-day mastectomy is safe, feasible, implicitly cost-effective, and likely associated with several potential advantages of self-care in the home setting. These results, supported by large, multi-institutional studies in 2 countries, show the success and utility of implementing a QI project and ERAS-driven pathway toward same-day mastectomy. Such a pathway, enhanced with a multimodal perioperative pain management regimen, can be potentially applied to implement a change in clinical practice from a hospital stay of 23 hours or beyond to an approximate 4- to 6-hour stay in the PACU and discharge to self-care at home. Future studies should focus on further refinement and optimization of pain management regimens, patient satisfaction, cost savings, and the eventual elimination of or need for oral opioid use in the postoperative home setting.

## Notes

This article meets the Accreditation Council for Graduate Medical Education and the American Board of Medical Specialties Maintenance of Certification competencies for Patient Care, Medical Knowledge, and Systems-Based Practice.
